# Adolescent idiopathic scoliosis research over the past 15 years: A bibliometric analysis of hotspots and emerging trends

**DOI:** 10.1097/MD.0000000000047469

**Published:** 2026-01-30

**Authors:** Renjie Hu, Shao Chen, Xiaomin Chen, Zhong Jiang, Honggen Du

**Affiliations:** a Department of Tuina, The First Affiliated Hospital of Zhejiang Chinese Medical University (Zhejiang Provincial Hospital of Chinese Medicine), Hangzhou, China.

**Keywords:** adolescent idiopathic scoliosis, bibliometric analysis, CiteSpace, emerging trends

## Abstract

**Purpose:**

To map the knowledge structure of adolescent idiopathic scoliosis (AIS) research from 2010 to 2024 and to identify emerging trends that are reshaping measurement, risk stratification, and intervention paradigms.

**Methods:**

Publications and reviews were retrieved from the Web of Science Core Collection (downloaded April 27, 2025). We quantified output and impact (citations, citations per publication, H-index) and visualized collaboration and intellectual structure using CiteSpace and Bibliometric.com. Networks were interpreted using centrality (bridging influence), co-citation clustering (knowledge base), and keyword burst/cluster analyses (emerging trends).

**Results:**

A total of 4924 records were included, with accelerated growth after 2017 and peak output in 2024. The USA led in impact and network connectivity, while China showed rapid expansion in volume with lower per-paper influence. Co-citation mapping revealed a transition from instrumentation- and mechanism-oriented foundations toward contemporary fronts in conservative management, growth modulation, genetic susceptibility, and data-driven assessment. Keyword bursts highlighted recent momentum in gene expression, intraoperative navigation, and machine learning, consistent with a broader shift toward precision-oriented and less invasive, decision-supportive care pathways.

**Conclusion:**

Adolescent idiopathic scoliosis research is undergoing a measurable thematic transition, in which emerging trends increasingly concentrate on quantification, prediction, and individualized management. These findings provide a field-level framework to prioritize multicenter collaboration, standardize outcomes, and accelerate translational evaluation of artificial intelligence–enabled assessment and risk models.

## 1. Introduction

Adolescent idiopathic scoliosis (AIS) is a spinal deformity distinguished by lateral curvature of the spine, occurring without underlying congenital or neuromuscular abnormalities.^[1]^ The reported prevalence of AIS varies across countries and regions, ranging from 0.47% to 5.2%.^[2]^ Although mild curvature may be asymptomatic, AIS can progress over time, resulting in musculoskeletal pain, cosmetic deformity, and, in severe cases, cardiopulmonary compromise.^[3]^ Growing evidence indicates that AIS is associated with significant psychological sequelae, including reduced self-esteem, body image disturbance, anxiety, and social withdrawal during adolescence.^[4,5]^ Altogether, these physical and psychosocial consequences make AIS a significant clinical and public health concern, highlighting the need for effective prevention, early detection, and individualized management strategies.

In recent years, AIS research has expanded rapidly across multiple disciplines. Beyond traditional investigations into epidemiology, curve progression, and surgical management, research priorities have broadened to encompass genetic susceptibility, molecular and endocrine mechanisms, biomechanical factors, and conservative treatment approaches such as bracing and exercise therapy. Emerging areas, including quantitative imaging assessment and artificial intelligence (AI)–assisted diagnosis and predictive modeling, have further diversified the field. While this expansion has substantially advanced knowledge of AIS, it has also increased the complexity of the literature, making it more challenging to systematically delineate the field’s overall developmental trajectory and emerging research frontiers.

Bibliometric analysis, recognized as an effective method for analyzing publication information, has been widely applied across various research domains.^[6–9]^ It involves the examination of published information, including books, journal articles, datasets, and their associated metadata, such as abstracts, keywords, and citations.^[10]^ This methodology employs statistical techniques to characterize relationships among published works, encompassing core scholars, institutions, and countries, along with their collaborative associations. It also includes analyses such as co-occurrence of keywords and co-citation patterns.^[11]^ Such an approach proves instrumental in unveiling the current state, popular topics, and continually evolving research trends within a specific field over time.

In recent years, several studies have applied bibliometric approaches to visualize and analyze the AIS research landscape. However, some limitations persist in the current body of work. Some analyses have focused narrowly on specific subdomains, such as surgical interventions,^[12]^ nonsurgical treatment,^[13]^ or etiological mechanisms,^[14]^ and therefore do not capture the field as a whole. Other studies have adopted broader perspectives but rely on datasets from earlier periods, limiting their ability to reflect recent paradigm shifts driven by advances in molecular biology, medical imaging, AI–assisted diagnostics, and evolving concepts of conservative management.^[15,16]^ To address these gaps, the present study conducts a comprehensive bibliometric analysis of AIS research published over the past 15 years, aiming to delineate established research hotspots and emerging trends and to provide updated insights to inform future investigations in this field.

## 2. Methods

### 2.1. Source of data and search strategy

Publications were retrieved from the Web of Science Core Collection (WoSCC) database using the following retrieval string: Topic = (idiopathic scoliosis) AND ((adolescent) OR (teenager) OR (juvenile)), with the limited time frame set from 2010 to 2024. To minimize bias resulting from daily database updates, all publication retrieval and data downloads were completed on April 27, 2025. We restricted the document types to original articles and reviews; conference abstracts, editorial materials, letters, and corrections were excluded during data retrieval.

### 2.2. Data collection and analysis

We conducted a retrieval from WoSCC, and all data retrieval was carried out by 2 authors. Before bibliometric analysis, duplicate records were systematically identified and removed. CiteSpace’s built-in de-duplication function was applied to identify and remove duplicate records based on bibliographic information, including title, authors, publication year, and source journal. This automated procedure was followed by manual inspection to eliminate residual near-duplicates caused by inconsistent metadata. Following de-duplication, 2 investigators independently screened titles and abstracts (and full texts when necessary) to ensure that the included records specifically addressed AIS (onset 10–18 years or explicitly described as adolescent). Studies primarily focusing on juvenile idiopathic scoliosis (onset 3–10 years), infantile idiopathic scoliosis, early-onset scoliosis, congenital/neuromuscular scoliosis, adult/degenerative scoliosis, or mixed cohorts without separable AIS data were excluded. Disagreements were resolved through discussion with a senior author. Given that the present analysis aimed to characterize research trends and knowledge structures rather than to synthesize clinical effect sizes, no formal study-level quality or risk-of-bias assessment was conducted.

Initially, we utilized WoSCC’s online analytical tools to analyze the retrieved data, obtaining various bibliometric parameters. These parameters included annual publication counts, country/region outputs, journals, total citation counts, citations per publication (CPP), and the H-index. The impact factors and quartiles for journal categories are sourced from the Journal Citation Reports 2023.

Additionally, we employed CiteSpace (version 6.2.R6) as our visualization analysis tool. All downloaded data were exported into CiteSpace (version 6.2.R6) for analyzing various essential bibliometric parameters, including co-citation analysis for countries, journals, institutions, authors, and references. This analysis also encompassed co-cited reference timeline views, capturing strong citation keywords and cluster maps for all items. The time slicing was set from 2010 to 2024 with a slice length of 1 year. Node selection in each slice was based on the g-index criterion, with the scaling factor *k* set to 8, 15, or 20 across different networks to balance network readability and representativeness, while other selection parameters were kept constant (link retaining factor [LRF] = 3.0, maximum links per node [L/N] = 10, look-back years [LBY] = 5, *e* = 1.0). No pruning algorithm was applied to preserve the full network structure, and 1.0% of nodes were labeled for visualization clarity. For cluster-based maps, Modularity Q and Weighted Mean Silhouette S reported by CiteSpace were used to evaluate the structural significance and within-cluster consistency of the clustering solutions, with detailed values presented in the corresponding figures. Furthermore, we utilized the bibliometrics website (http://bibliometric.com/) to generate a cooperative analysis graph for countries/regions.

## 3. Results

### 3.1. Annual number of publications and citations

**Figure 1.**
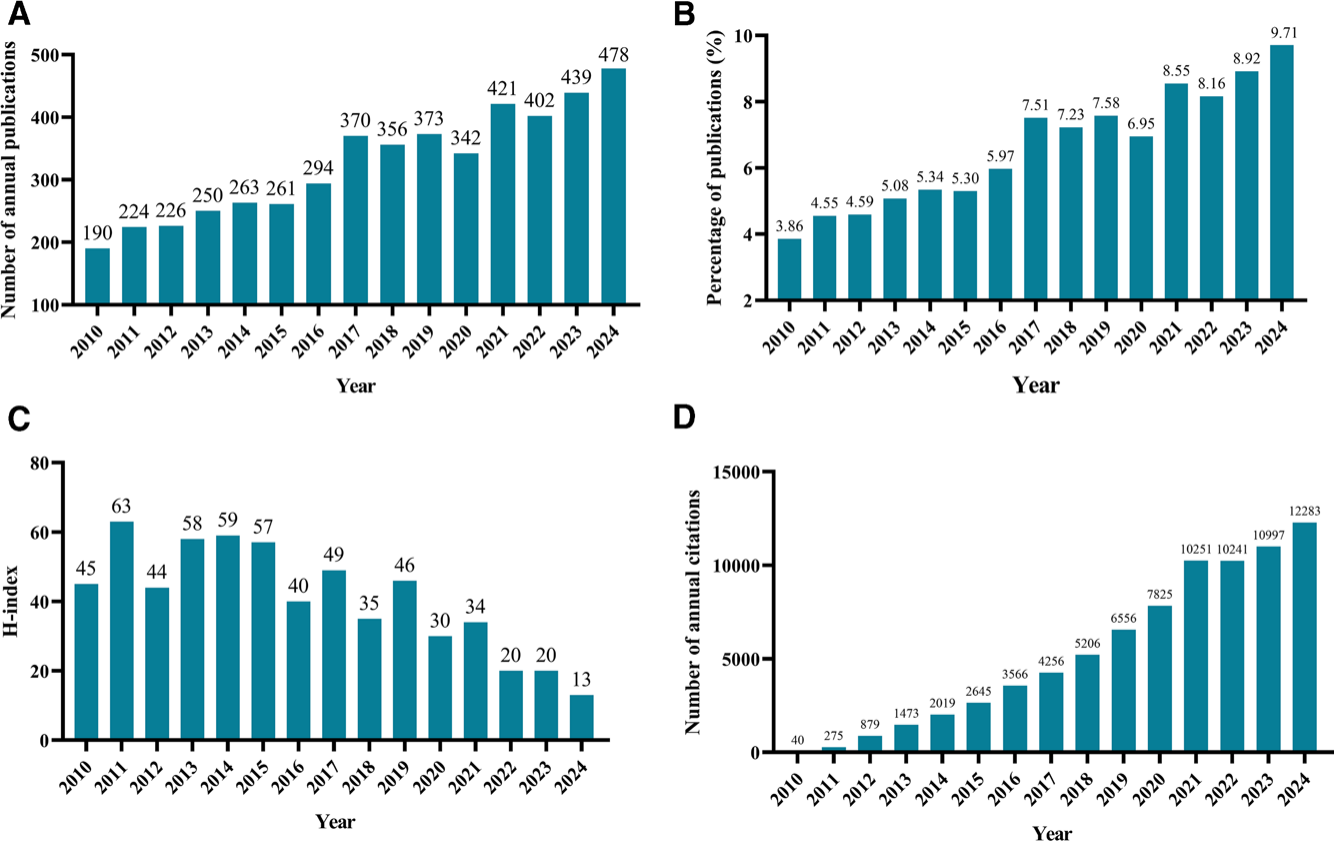
(A) The number of publications. (B) The annual publication count as a proportion of the total number of publications. (C) The H-index for publications each year. (D) The number of annual citations.

The bibliometric analysis encompassed 4924 publications published between 2010 and 2024, comprising 4433 articles and 491 reviews. Figure 1 illustrates the characteristics of these publications, reflecting the overall development and academic impact of the field. As depicted in Figure 1A and B, there has been a significant increase in annual publication numbers since 2017, reaching its peak in 2024 (*N* = 478, accounting for 9.71% of the total). This pronounced growth indicates a rapid expansion of research activity and suggests that the topic has attracted increasing scholarly attention in recent years.

The temporal evolution of the H-index is illustrated in Figure 1C. The H-index reached its highest value in 2011, followed by a relatively stable phase and a gradual decline after 2015. This pattern can be partly attributed to the time-dependent nature of citation accumulation, as publications from recent years have had less time to accrue citations, rather than indicating a reduction in research quality or influence.

In terms of citation performance, the collected publications had received a total of 82,141 citations as of the retrieval date, corresponding to a citations per publication (CPP) of 16.68 and an overall H-index of 95. As shown in Figure 1D, annual citations increased substantially over time, with the highest citation counts observed in 2024 (12,283 citations), followed by 2023 (10,997 citations) and 2021 (10,251 citations). The sharp rise in recent annual citations highlights the growing academic visibility and influence of the field, consistent with the observed surge in publication output.

### 3.2. Distribution of countries/regions and institutions

**Figure 2.**
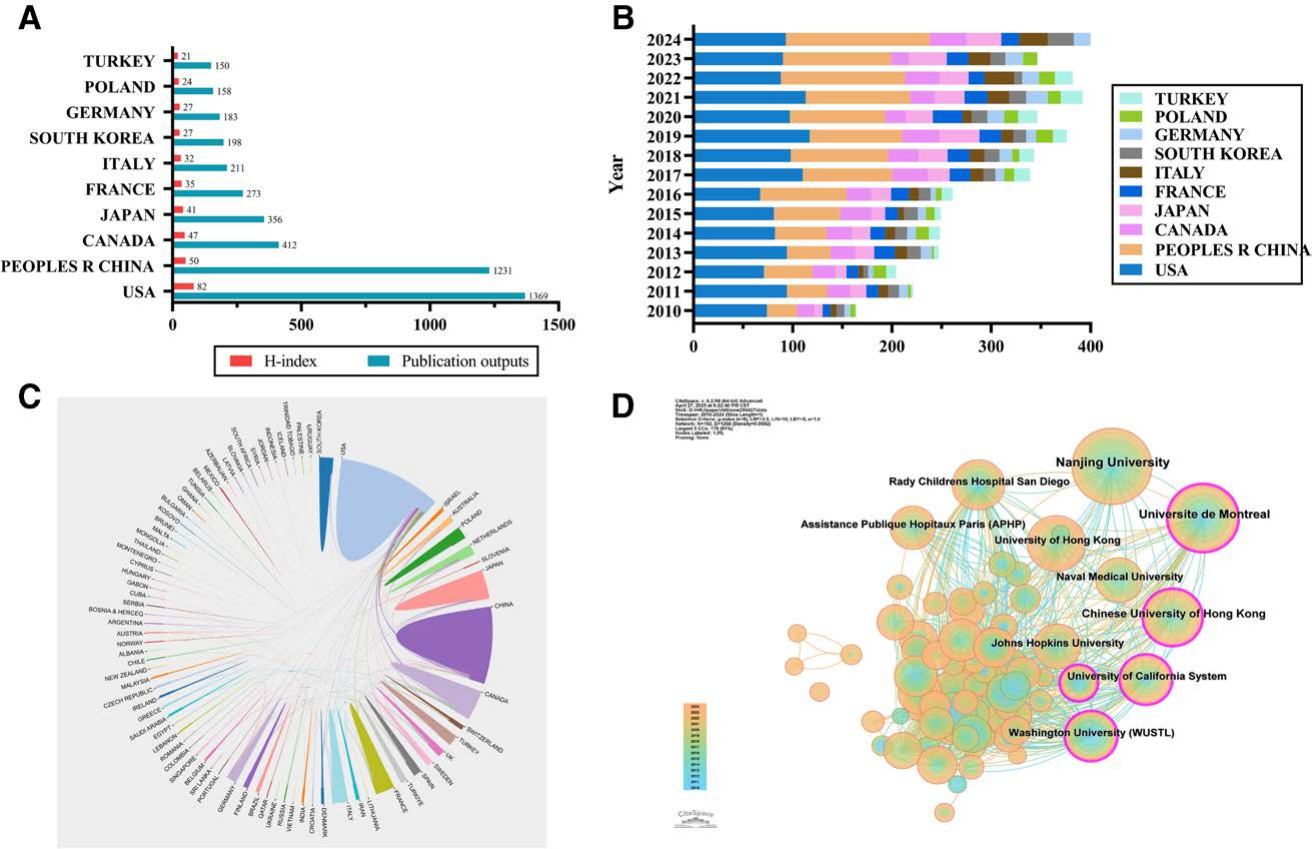
(A) The number of publications and H-index of the top 10 countries/regions with the highest number of published articles. (B) The annual publication counts for the top 10 countries/regions with the highest number of published articles. (C) The international collaboration map between different countries. (D) The collaboration map among institutions in the field of AIS. In the collaboration networks, nodes represent countries/regions or institutions, and node size reflects publication volume. Links indicate collaborative relationships. Node colors correspond to different publication years (time slices), ranging from earlier years (cool colors) to more recent years (warm colors). Nodes surrounded by a purple outer ring indicate high betweenness centrality. AIS, adolescent idiopathic scoliosis.

The analyzed publications originated from 88 countries and regions, reflecting the global scope of research in the AIS field. Table 1A outlines the top 10 countries/regions with the highest number of published articles. As shown in Table 1 and visualized in Figure 2A, the USA leads with the highest number of publications (27.80%, totaling 1369 articles), followed by China (25.00%, with 1231 articles), Canada (8.37%, with 412 articles), Japan (7.23%, with 356 articles), and France (5.54%, with 273 articles). Notably, the USA also exhibited the highest H-index (82) and a high CPP (24.31), indicating both sustained productivity and strong academic impact. In comparison, although China demonstrated comparable publication volume, its CPP (14.08) and H-index (50) were relatively lower, suggesting differences in citation impact among leading countries. Overall, Western countries such as the USA, Canada, and several European nations tended to show higher CPP values, reflecting greater average citation influence.

**Table 1:** Leading countries/regions and institutions contributing to adolescent idiopathic scoliosis research.

(A) Countries/regions (top 10)												
Rank		Countries/Regions		Record count			% of 4924			H-index	CPP	
1		USA		1369			27.80			82	24.31	
2		People’s R China		1231			25.00			50	14.08	
3		Canada		412			8.37			47	23.61	
4		Japan		356			7.23			41	18.03	
5		France		273			5.54			35	17.51	
6		Italy		211			4.29			32	17.86	
7		South Korea		198			4.02			27	15.19	
8		Germany		183			3.72			27	16.93	
9		Poland		158			3.21			24	12.84	
10		Turkey		150			3.05			21	12.47	

(B) Institutions (top 10)												
Rank	Institution		Count		% of 4924	CPP		H-index	Location			Centrality
1	Nanjing University		252		5.12	17.86		34	China			0.08
2	University de Montreal		189		3.84	20.84		32	Canada			0.11
3	The Chinese University of Hong Kong		155		3.15	21.08		30	China (Hong Kong SAR)			0.1
4	The University of Hong Kong		125		2.54	22.02		30	China (Hong Kong SAR)			0.09
5	University of California System		122		2.48	37.64		36	USA			0.12
6	Rady Children’s Hospital – San Diego		113		2.29	27.98		31	USA			0.07
7	Naval Medical University		111		2.25	14.35		20	China			0.06
8	Assistance Publique–Hôpitaux de Paris		103		2.09	17.16		25	France			0.09
9	Johns Hopkins University		100		2.03	25.85		25	USA			0.08
10	Washington University in St. Louis		95		1.93	43.21		32	USA			0.16

CPP = citations per publication.

The temporal trends in annual publication outputs of the top 10 countries/regions are illustrated in Figure 2B. Throughout the study period, the USA and China consistently occupied the top 2 positions in annual publication output. While the USA maintained a relatively stable level of annual productivity, China displayed a pronounced growth trend despite a temporary decline in 2021. Since 2022, China has surpassed the USA in annual publication counts, indicating a rapid expansion of research activity and increasing scholarly engagement in AIS research.

International collaboration patterns among countries/regions are depicted in Figure 2C. In this collaboration network, nodes represent countries/regions, and links indicate co-authorship relationships between them, with thicker links reflecting stronger collaborative intensity. The USA occupies a central position in the network, forming extensive collaborations with multiple countries. Particularly strong collaborative ties were observed between the USA and Canada, followed by China and Japan, highlighting the pivotal role of the USA as a global hub facilitating international research collaboration in this field.

At the institutional level, a total of 192 institutions contributed to AIS-related publications. The top 10 institutions by publication output are listed in Table 1B, which details their publication counts, CPP, H-index values, geographic locations, and betweenness centrality scores. As shown in Figure 2D, the most productive institutions included Nanjing University (252 publications, 5.12%), University of Montreal (189 publications, 3.84%), The Chinese University of Hong Kong (155 publications, 3.15%), The University of Hong Kong (125 publications, 2.54%), and the University of California System (122 publications, 2.48%). The institutional collaboration network consists of 192 nodes and 1268 links, where node size reflects publication output and link represents collaborative relationships.

Although Nanjing University ranked first in publication output, it did not exhibit the highest CPP (17.86) or centrality (0.08). In contrast, the University of California System and Washington University in St. Louis showed relatively high CPP values (37.64 and 43.21, respectively) and higher betweenness centrality, indicating greater academic influence and more prominent bridging roles within the collaboration network. Notably, the University of Montreal also demonstrated high centrality (0.11), highlighting its importance in connecting multiple institutional clusters.

### 3.3. Distribution of journals

A total of 995 scholarly journals published articles related to AIS research during the study period. The top 10 most productive journals are summarized in Table 2A, which presents detailed information on publication output, citation performance, impact indicators, and journal characteristics. Among them, Spine emerged as the leading journal, publishing 690 articles and accounting for 14.01% of all included publications. In addition to its high productivity, Spine also demonstrated substantial academic influence, with the highest H-index (62) and a relatively high CPP (27.5), confirming its central role in disseminating AIS research.

**Table 2:** Core publication venues and influential contributors in adolescent idiopathic scoliosis research.

(A) Journals (top 10 by record count)																	
Rank	Journal		Count		% of 4924		CPP	H-index		IF (2023)		JCI (2023)	Total citations		Country	Category quartile	
1	*Spine*		690		14.01		27.5	62		2.7		1.14	18,949		USA	Q1	
2	*European Spine Journal*		603		12.25		20	52		2.6		0.93	12,050		USA	Q2	
3	*Journal of Pediatric Orthopaedics*		193		3.92		16.3	29		1.4		0.73	3145		USA	Q2	
4	*Spine Journal*		166		3.37		23.9	35		4.9		1.7	3964		USA	Q1	
5	*BMC Musculoskeletal Disorders*		129		2.62		10.3	20		2.2		0.82	1327		UK	Q2	
6	*World Neurosurgery*		104		2.11		8.3	15		1.9		0.68	863		USA	Q3	
7	*Journal of Clinical Medicine*		95		1.93		3.97	10		3.0		0.92	377		USA	Q1	
8	*Journal of Bone and Joint Surgery* (American volume)		85		1.73		33.5	30		4.4		2.11	2844		USA	Q1	
9	*PLoS One*		80		1.62		16.5	23		2.9		0.88	1321		USA	Q1	
10	*Global Spine Journal*		79		1.60		5.53	11		2.6		0.86	437		Germany	Q2	

(B) Authors and co-cited authors (top 10)																	
Rank		Author		Count		CPP			H-index		Rank			Co-cited author			Count
1		Qiu, Yong		222		17.03			31		1			Weinstein S. L.			1285
2		Zhu, Zezhang		145		13.11			24		2			Lenke L. G.			1152
3		Newton, PO		101		31.26			31		3			Kim Y. J.			651
4		Liu, Zhen		96		16.44			23		4			Suk S. I.			648
5		Parent, Stefan		87		19.48			26		5			Negrini S.			585
6		Labelle, Hubert		76		23.28			26		6			Newton P. O.			533
7		Li, Ming		68		14.77			18		7			Lonstein J. E.			507
8		Xu, Leilei		61		16.37			18		8			Stokes I. A. F.			453
9		Samdani, Amer F.		56		24.4			25		9			Danielsson A. J.			406
10		Shah, Suken A.		53		24.68			22		10			Richards B. S.			398

CPP = citations per publication, IF = impact factor, JCI = Journal Citation Indicator.

Following Spine, the *European Spine Journal* ranked second with 603 publications (12.25%), along with a high H-index (52), indicating its sustained contribution to the field. Other prominent journals included the *Journal of Pediatric Orthopaedics* (193 publications), *Spine Journal* (166 publications), and *BMC Musculoskeletal Disorders* (129 publications). Notably, although *Spine Journal* ranked fourth in publication volume, it exhibited the highest impact factor (4.9) and a high Journal Citation Indicator (1.7), reflecting strong citation performance and journal prestige. In contrast, the *Journal of Bone and Joint Surgery* (American volume) demonstrated the highest CPP (33.5) among the top 10 journals, despite a comparatively lower publication count, suggesting that articles published in this journal tend to receive higher average citation impact.

From a geographical perspective, 7 out of the top 10 journals were published in the USA, underscoring the dominant role of US-based journals in shaping and disseminating AIS research. Furthermore, most of these leading journals were ranked in the Q1 or Q2 quartiles within their respective categories, indicating overall high academic quality and visibility.

### 3.4. Distribution of authors and cited authors

A total of 453 authors contributed to AIS-related publications during the study period. The top 10 most productive authors are listed in Table 2B, together with their publication counts, citations per publication (CPP), and H-index values. Among them, Qiu, Yong ranked first with 222 publications, followed by Zhu, Zezhang (145 publications), Newton, Peter O. (101 publications), Liu, Zhen (96 publications), and Parent, Stefan (87 publications). These authors represent the most active contributors driving research output in the AIS field.

**Figure 3.**
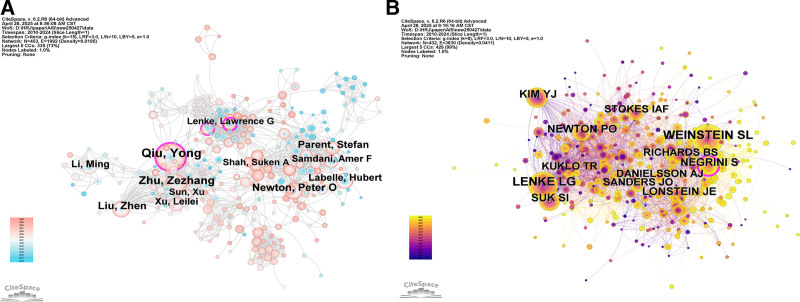
(A) Author contribution network in the field of AIS. (B) The map of co-cited authors identified across all publications. In both networks, nodes represent authors. In (A), node size reflects authors’ publication output, whereas in (B), node size represents co-citation frequency. Links indicate collaboration (A) or co-citation relationships (B). Node colors indicate different publication years (time slices). A purple outer ring denotes high betweenness centrality. AIS, adolescent idiopathic scoliosis.

The author collaboration network is visualized in Figure 3A, where nodes represent authors and links indicate co-authorship relationships, with larger nodes corresponding to higher publication output. The network reveals several tightly connected collaborative clusters, suggesting the presence of stable research teams and long-term partnerships. Authors such as Qiu, Yong, Zhu, Zezhang, and Newton, Peter O. occupy prominent positions in the network, reflecting their central roles in coordinating collaborative research activities.

In contrast to productive authorship, the co-cited author network shown in Figure 3B reflects the intellectual foundation of AIS research. The top 10 co-cited authors are also summarized in Table 2B. Weinstein, S. L. ranked first with 1285 co-citations, followed by Lenke, L. G. (1152 co-citations), Kim, Y. J. (651 co-citations), Suk, S. I. (648 co-citations), and Negrini, S. (585 co-citations). These highly co-cited authors constitute the core knowledge base of the field, as their works are frequently cited together and provide foundational theoretical and clinical frameworks for subsequent studies.

Notably, Newton, Peter O. appears in both rankings, demonstrating a dual role as a highly productive author and a highly influential contributor. Although ranking sixth among co-cited authors (533 co-citations), Newton exhibited the highest CPP (31.26) and shared the highest H-index (31) among the top productive authors, indicating both strong research impact and sustained academic influence. This overlap highlights the distinction yet potential convergence between research productivity and intellectual influence, suggesting that while many authors actively contribute to the literature, only a subset simultaneously shapes the foundational knowledge structure of AIS research.

### 3.5. Distribution of cited references

**Figure 4.**
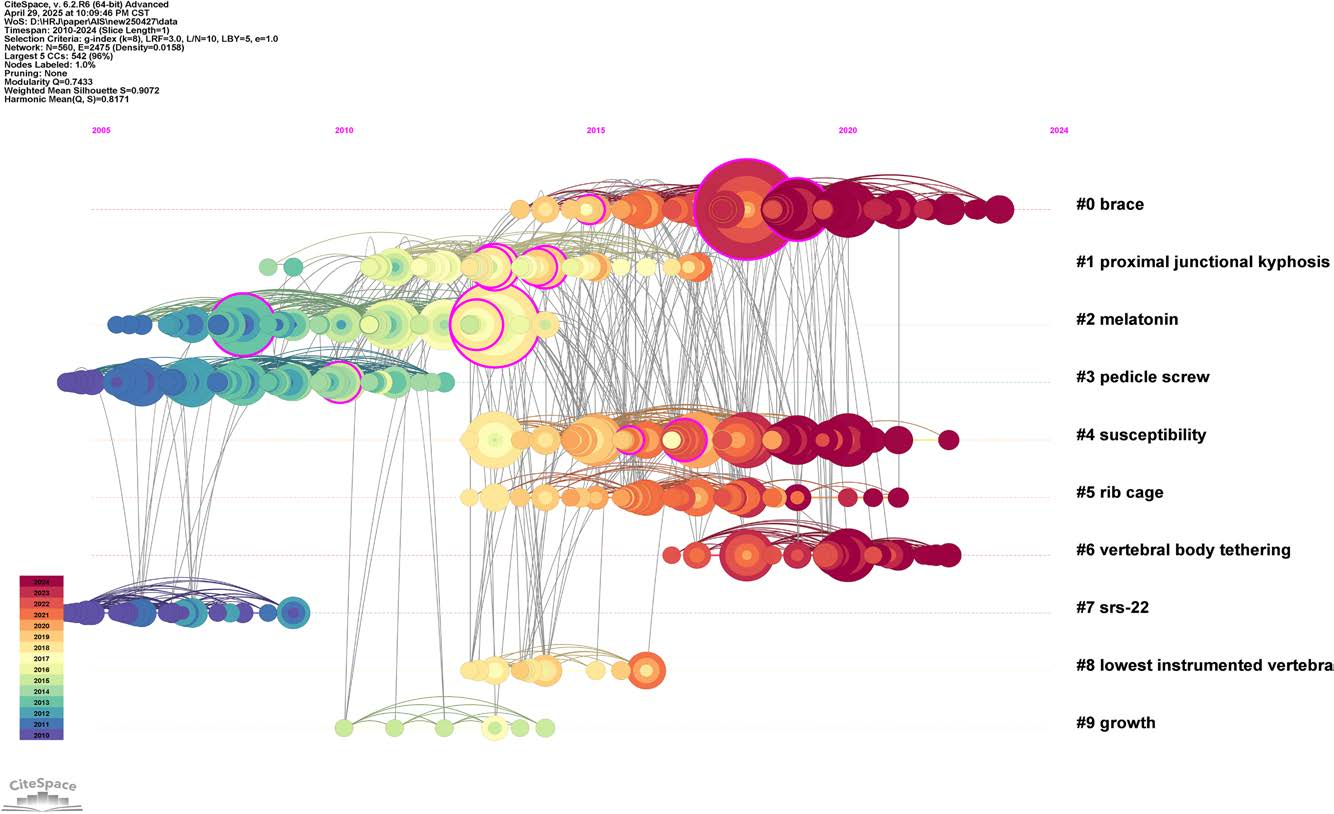
The timeline view of co-cited references related to AIS research. In this visualization, clusters are displayed horizontally along timelines, and the label of each cluster is shown at the end of the corresponding timeline. Nodes represent cited references, and node size represents co-citation frequency. Links indicate co-citation relationships between references. References are arranged along the horizontal timeline according to their time information in the co-citation network, and seminal references may appear earlier than the literature retrieval window. Node colors (tree rings) reflect temporal information, ranging from earlier years (cool colors) to more recent years (warm colors). AIS, adolescent idiopathic scoliosis.

The timeline view of co-cited references related to AIS research is presented in Figure 4, providing a dynamic visualization of the intellectual structure and temporal evolution of the field. The clustering was performed using the log-likelihood ratio algorithm, which identifies representative terms for each cluster based on citation patterns. In the timeline visualization, each node represents a co-cited reference, with larger nodes indicating higher co-citation frequency, while node colors correspond to different time slices, where warmer colors denote more recent publications.

A total of 10 major co-citation clusters were identified, reflecting distinct research themes and developmental stages within AIS research. As summarized in Table 3, each cluster is characterized by its size, average publication year, silhouette score, and log-likelihood ratio-derived label. The silhouette scores of all major clusters exceeded 0.80, indicating high clustering quality and strong internal consistency. Among them, Cluster #9 (“growth”) exhibited the highest silhouette value (0.989), suggesting an especially coherent thematic structure.

**Table 3:** The structural characteristics of the co-citation clusters.

Cluster label	Number of nodes	Silhouette score	Mean year	Keyword (LLR)
#0	95	0.859	2018	Brace
#1	86	0.861	2013	Proximal junctional kyphosis
#2	83	0.961	2009	Melatonin
#3	78	0.947	2008	Pedicle screw
#4	61	0.855	2016	Susceptibility
#5	43	0.884	2016	Rib cage
#6	33	0.985	2020	Vertebral body tethering
#7	31	0.982	2006	SRS-22
#8	16	0.965	2014	Lowest instrumented vertebra
#9	10	0.989	2011	Growth

LLR = log-likelihood ratio.

From a temporal perspective, Cluster #0 (“bracing”) was the largest cluster, comprising 95 nodes with a mean publication year of 2018, indicating its long-standing and sustained importance in AIS research. Other relatively early clusters, such as Cluster #2 (“melatonin,” mean year 2009) and Cluster #3 (“pedicle screw,” mean year 2008), reflect foundational research focusing on etiological mechanisms and surgical instrumentation. In contrast, several clusters emerged more recently, highlighting evolving research frontiers. Notably, Cluster #6 (“vertebral body tethering,” mean year 2020) represents a rapidly developing topic related to growth-modulation techniques, while Cluster #4 (“susceptibility,” mean year 2016) and Cluster #5 (“rib cage,” mean year 2016) reflect increasing interest in genetic factors and biomechanical aspects of AIS.

Overall, the timeline view reveals a clear transition in AIS research from early emphases on surgical techniques and biological mechanisms toward more recent focuses on conservative-management strategies, growth modulation, and individualized risk assessment. The combination of high silhouette scores and distinct temporal patterns across clusters demonstrates a well-structured knowledge base and highlights the dynamic evolution of research hotspots in the AIS field.

### 3.6. Distribution of keywords burst

A total of 595 keywords were extracted from the included publications using CiteSpace. The top 20 most frequently occurring keywords are summarized in Table 4, reflecting the core research topics in AIS over the past 15 years. As expected, “AIS” and “idiopathic scoliosis” ranked first and second, respectively. Excluding these disease-specific terms, “surgery” emerged as the most frequent keyword (frequency = 567), followed by related terms such as “fusion,” “internal fixation,” and “posterior spinal fusion.” This pattern highlights the long-standing dominance of surgical management as a central focus of AIS research. In addition, keywords such as “quality of life,” “curve progression,” and “treatment outcome” indicate increasing attention to patient-centered outcomes and long-term clinical effectiveness.

**Table 4:** Top 20 frequency of keywords in adolescent idiopathic scoliosis research.

Rank	Keywords	Frequency	Rank	Keywords	Frequency
1	Adolescent idiopathic scoliosis	2991	11	Reliability	329
2	Idiopathic scoliosis	655	12	Pedicle screw	296
3	Surgery	567	13	Spinal fusion	271
4	Fusion	533	14	Curve progression	269
5	Internal fixation	512	15	Instrumentation	258
6	Children	438	16	Treatment outcome	258
7	Posterior spinal fusion	387	17	Complication	250
8	Spinal deformity	365	18	Spinal malformation	240
9	Quality of life	352	19	Surgical treatment	236
10	Spine	348	20	Follow-up	232

**Figure 5.**
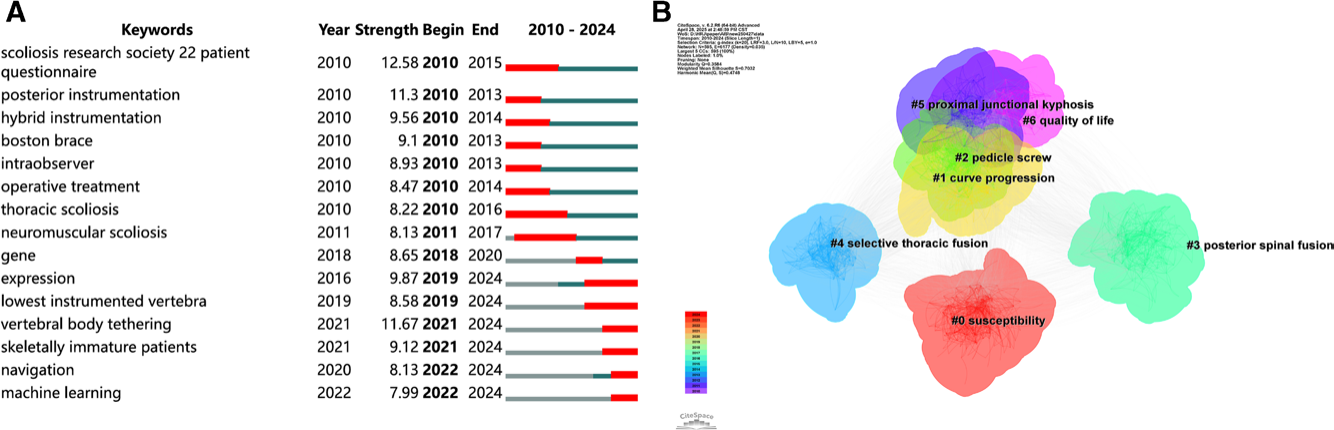
(A) The map of burst strength rank of keywords from 2010 to 2024. (B) The map of cluster analysis of emerging trends in keywords from 2010 to 2024. In panel (B), nodes represent keywords, and node size reflects keyword frequency. Links indicate co-occurrence relationships between keywords. Node colors correspond to different publication years (time slices), ranging from earlier (cool colors) to more recent years (warm colors).

To identify established research hotspots and emerging trends, keyword burst detection was performed, and the results are visualized in Figure 5A. The keyword with the strongest burst strength was “Scoliosis Research Society-22 Patient Questionnaire” (burst strength = 12.58), underscoring the growing emphasis on standardized outcome assessment and health-related quality of life evaluation. Notably, several burst keywords have appeared predominantly in the most recent 5 years, including “gene expression,” “lowest instrumented vertebra,” “vertebral tethering,” “skeletally immature patients,” “intraoperative navigation,” and “machine learning.” These emerging terms suggest a shift in research focus toward genetic mechanisms, growth-modulation strategies, precision surgical planning, and data-driven clinical decision support. However, from a structural perspective of the keyword network, these emerging terms are mainly concentrated in relatively small and temporally recent clusters and show limited overlap with the largest and most central clusters, such as “bracing” and “curve progression.” In addition, the burst keywords related to machine learning and genetic susceptibility exhibit strong temporal novelty but relatively weak structural centrality, indicating that these topics, although rapidly developing, have not yet been fully integrated into the core knowledge framework of AIS research.

Furthermore, keyword clustering analysis was conducted to reveal the thematic structure of recent AIS research, as shown in Figure 5B. Seven major clusters were identified, labeled as “susceptibility,” “curve progression,” “pedicle screws,” “posterior spinal fusion,” “selective thoracic fusion,” “proximal junctional kyphosis,” and “quality of life.” Collectively, these clusters encompass etiological research, deformity progression, surgical techniques, complication management, and patient-reported outcomes, demonstrating a comprehensive and multidimensional research landscape. The coexistence of surgically oriented clusters and outcome-focused clusters further indicates a gradual transition from technique-centered studies toward individualized treatment strategies and long-term outcome optimization in AIS research.

## 4. Discussion

### 4.1. Overall evolution of AIS research and main findings

This bibliometric study set out to delineate how AIS research has evolved over the past 15 years and, in particular, to identify the intellectual base, collaborative structure, and emerging thematic fronts. Taken together, the results depict a field that has expanded rapidly in volume and breadth, while its conceptual center has begun to shift – from established research hotspots in conservative management toward emerging trends in precision-oriented stratification, growth-modulation approaches, and data-driven assessment workflows.

The current analysis indicates a growing trend in both the annual publication count and citation frequency in the field of AIS, reaching its peak in 2024. One way to understand the marked growth in AIS publications is to see it less as a simple rise in interest and more as a sign of disciplinary convergence. Orthopaedics remains the core, but contemporary AIS questions increasingly recruit methods and concepts from genetics, biomechanics, imaging science, and computational modeling. From this perspective, the recent prominence of analytics-heavy themes is not a transient trend; it reflects the practical reality that AIS management hinges on probabilistic decisions (progression risk, timing of intervention, and compliance-sensitive outcomes) and therefore benefits from tools that improve prediction, measurement reliability, and individualized care pathways.

### 4.2. Geographic, institutional, and authorship structure of AIS research

The geographic and institutional topology further clarifies where the field’s momentum is concentrated. The USA continues to occupy a central position in impact and network connectivity, whereas China shows fast output growth but comparatively weaker indicators of bridging centrality and per-paper influence. This “output–influence” asymmetry is best interpreted cautiously, yet it aligns with familiar structural determinants of high-impact production: sustained multicentre cohorts, standardized registries, and trial infrastructure tend to yield work that is both reusable and widely cited. Conversely, rapid output expansion can be accompanied by fragmentation – heterogeneous endpoints, single-center samples, and limited integration into multinational programs – which dilutes visibility even when the underlying clinical questions are similar. In practical terms, the data imply that the next step for high-output regions is not simply more papers but stronger connective tissue: shared protocols, interoperable datasets, and collaborative prospective studies that are explicitly designed for generalizability.

The author and co-citation architecture offer a complementary lens on field maturation. The pattern – stable, highly co-cited knowledge anchors alongside prolific producers – suggests that AIS research is propelled by a small set of widely reused conceptual and evidentiary resources (trials, consensus statements, and foundational etiologic frameworks), while thematic production continues to diversify at the periphery. Put differently, the field’s direction is increasingly shaped by “infrastructure publications” that standardize definitions, methods, and outcomes, which then get imported into adjacent subdomains such as prediction modeling, growth modulation, and technology-assisted monitoring.

### 4.3. Thematic transition and emerging research fronts in AIS

The clearest signal of thematic transition emerges from keyword co-occurrence and burst dynamics. Classic AIS research hotspots remain durable – bracing, curve progression, biomechanics, and clinically relevant outcomes – yet recent bursts emphasize emerging trends such as vertebral body tethering, skeletally immature patients, intraoperative navigation, and machine learning.

Set against high-impact literature, these bibliometric patterns are largely coherent and, importantly, help sharpen what is novel about the present study. The continuing prominence of bracing-related themes is consistent with trial-level evidence showing that bracing reduces progression to surgical thresholds and with systematic syntheses supporting effectiveness under defined indications.^[17]^ Exercise-based approaches, while increasingly discussed, still sit in a space where evidence quality, protocols, and uptake vary meaningfully across settings.^[18,19]^ Notably, the rise of keywords around risk factors, compliance, and treatment failure fits with a broader reframing in AIS: the question has moved beyond whether bracing works in principle to when it works in practice, and for whom, given behavioral and biomechanical constraints.^[20,21]^

The growing visibility of genetic susceptibility themes also aligns with the consolidation of AIS as a multifactorial disorder with reproducible genetic associations.^[22,23]^ Here, an important interpretive nuance is that bibliometric prominence does not imply etiologic closure; rather, it signals that genetics is increasingly being treated as a translational input – something that can inform stratification, enrich trials, and support risk modeling. Recent work on polygenic scoring underscores this direction and illustrates how susceptibility signals might be repurposed into clinically meaningful risk estimates.^[24]^ Likewise, the emergence of vertebral tethering in frontier clusters resonates with a growing clinical and technical discourse around non-fusion growth modulation, including early conceptual demonstrations and evolving surgical technique work.^[25,26]^ Finally, the rise of machine learning and automated measurement themes is increasingly supported by recent evidence across multiple acquisition modes. For radiographs, deep-learning systems have demonstrated automated Cobb angle measurement with strong agreement relative to clinicians, supporting efficiency and reproducibility gains in routine follow-up.^[27,28]^ Beyond 2D coronal angles, AIS is inherently a 3D deformity, and recent work has emphasized 3D-informed predictors and reconstruction from biplanar imaging, aligning with the growing bibliometric visibility of imaging science and computational modeling.^[29,30]^ In parallel, photo-based or low-burden monitoring has advanced toward clinically oriented classification and longitudinal monitoring with validated deep-learning approaches, reinforcing workflow innovation as a near-term translational pathway.^[31]^ In aggregate, the present study’s contribution is not simply that AIS research is increasing, but that it specifies how the field’s center of gravity is moving – toward integrated pipelines that connect stratification, scalable monitoring, and less invasive interventions – while the conservative-management evidence base remains structurally central.

### 4.4. Academic and translational implications for AIS research and care

These observations carry implications at both academic and translational levels. From a theory-building standpoint, the field appears to be converging on a pragmatic synthesis: etiologic and mechanistic questions (genetic architecture, neuromuscular and endocrine hypotheses, and biomechanical progression models) increasingly matter insofar as they can be coupled to prediction, surveillance, and treatment selection. Clinically, the prominence of automation and machine learning bursts suggests a near-term translational pathway that is often underestimated – workflow innovation. Standardized, low-burden monitoring tools that are transparently validated across settings could plausibly improve care quality even before deeper etiologic mechanisms are fully resolved.

### 4.5. Comparison with previous bibliometric analyses and updated field trajectory

Compared with previous bibliometric analyses of scoliosis or AIS, the present study offers a substantively updated and more interpretive account of how the field has evolved in its most recent phase. Our findings are broadly concordant with earlier bibliometric mappings of scoliosis/AIS research in 2 respects: continued expansion of the literature and a persistent concentration of output within a small set of countries, institutions, and spine-focused journals.^[15,16,32]^ These convergent signals strengthen the interpretation that AIS research has matured into a high-throughput, internationally distributed field, but one still shaped by a relatively stable set of infrastructural venues and collaborative nuclei.

Where our results diverge from, and extend beyond, prior studies is in the nature of the field’s thematic transition and the granularity with which it can be interpreted. Methodologically, the earlier analyses were optimized for different purposes: Tao et al^[16]^ combined Web of Science with PubMed MeSH term co-word biclustering (BICOMB/gCLUTO) to define 8 scoliosis “hotspots” and explicitly predicted that AI diagnosis and genetic research would become future foci. Zhao et al^[15]^ used VOSviewer keyword co-occurrence to cluster AIS research into 4 major thematic blocks – surgical treatment/complications, conservative therapy/progression, alignment/classification, and etiology/pathogenesis – and showed an evolution from instrumentation/natural-history/bracing-centric keywords toward “outcomes,” “prevalence,” and “genome association.” Guler et al^[32]^ deliberately adopted a broad “scoliosis” strategy and focused on global output, citation structure, and trend-topic mapping across all scoliosis phenotypes (1980–2019), necessarily blending pediatric idiopathic scoliosis with adult deformity and other etiologic categories. In contrast, by restricting scope to AIS and explicitly interrogating the intellectual base (co-citation architecture), collaborative topology, and emerging thematic fronts over a recent 15-year window, the present study is positioned to move beyond cataloguing “what is popular” toward clarifying how the field’s center of gravity is relocating – from consolidation of conservative-management evidence to precision-oriented stratification, growth-modulation strategies, and data-driven assessment pipelines. This design also helps explain why certain signals appear attenuated or amplified relative to prior work: broader scoliosis searches tend to elevate adult alignment/classification and degenerative deformity themes, whereas an AIS-restricted corpus sharpens the visibility of growth-linked intervention logics and pediatric monitoring workflows.

Importantly, our thematic conclusions both validate and update the trajectories implied by earlier bibliometrics. Tao et al’s forward-looking claim that AI-enabled diagnosis and genetics would become hotspots is now consistent with the contemporary prominence of analytics-heavy themes and genetic susceptibility framing observed in AIS-focused mapping, where “genome association” rose among recent keywords in Zhao et al’s analysis. Likewise, the persistence of bracing/progression as a durable knowledge anchor in Zhao et al’s conservative therapy cluster aligns with our observation that conservative management remains structurally central even as innovation migrates toward individualized risk prediction, scalable longitudinal monitoring, and less invasive growth-modulating interventions. Taken together, the comparison suggests a coherent field-level narrative: the AIS literature is not simply expanding; it is reorganizing around an integrated translational loop in which mechanistic signals (e.g., genetics) increasingly serve stratification and prediction, while technology (automation/ML) increasingly serves measurement reliability, surveillance scalability, and workflow feasibility.

## 5. Limitations

While this study diligently adheres to the principles of bibliometric analysis, several inevitable limitations should be acknowledged. First, using a single bibliographic database necessarily constrains coverage: regional journals, non-indexed outputs, and conference-first innovations may be underrepresented, which can bias geographic comparisons and delay early signals for emerging techniques. Second, citation-based influence is time-dependent and systematically disadvantages newer work; consequently, frontier topics identified via bursts might not yet appear as dominant in co-citation structures. Third, clustering outcomes depend on parameterization and the stability of keyword/reference metadata; alternative thresholds can change cluster granularity even when the overall direction remains stable. Finally, bibliometric prominence reflects attention and connectivity, not clinical efficacy or mechanistic truth. In other words, these results should guide how we understand research structure and opportunity, but they cannot replace critical appraisal of evidence quality.

## 6. Conclusion

AIS research over the past 15 years has undergone a clear thematic transition from established research hotspots centered on conservative management toward emerging trends in precision stratification, growth-modulation strategies, and scalable data-driven assessment. As demonstrated by our bibliometric findings – specifically, recent keyword bursts in machine learning, intraoperative navigation, and genetic susceptibility, together with collaboration network patterns showing rapid output growth but uneven cross-center influence – future research priorities can be directly inferred from the field’s structural and thematic evolution. These priorities include prospective, multicenter evaluation and external validation of AI-driven diagnostic and monitoring tools in response to the growing prominence of analytics-based assessment themes; registry-enabled multicenter collaborations with standardized outcomes to address fragmentation and improve publication quality highlighted by the observed output-influence asymmetry; genetic risk modeling, including polygenic approaches, integrated with longitudinal clinical and imaging trajectories, reflecting the consolidation of susceptibility-related clusters and their translational potential for individualized risk stratification and treatment selection; and comparative-effectiveness and decision-impact studies to determine whether emerging technologies warrant integration into core management algorithms.

## Acknowledgments

The authors would like to convey their appreciation to Professor Chaomei Chen for creating and sharing the CiteSpace software.

## Author contributions

**Conceptualization:** Honggen Du.

**Data curation:** Renjie Hu, Shao Chen.

**Supervision:** Zhong Jiang.

**Writing – original draft:** Renjie Hu.

**Writing – review & editing:** Renjie Hu, Shao Chen, Xiaomin Chen, Zhong Jiang, Honggen Du.
